# Targeting non-coding RNAs in unstable atherosclerotic plaques: Mechanism, regulation, possibilities, and limitations

**DOI:** 10.7150/ijbs.62506

**Published:** 2021-08-03

**Authors:** Xiaoxin Li, Yanyan Yang, Zhibin Wang, Shaoyan Jiang, Yuanyuan Meng, Xiaoxia Song, Liang Zhao, Lu Zou, Min Li, Tao Yu

**Affiliations:** 1 Institute for translational medicine, The Affiliated Hospital of Qingdao University, No. 38 Dengzhou Road, 266021, People's Republic of China.; 2 Department of Cardiac Ultrasound, The Affiliated Hospital of Qingdao University, Qingdao 266000, China.; 3 Department of Cardiology, The Affiliated Cardiovascular Hospital of Qingdao University, No. 5 Zhiquan Road, Qingdao 266000, China.

**Keywords:** atherosclerotic plaques, plaque instability, plaque rupture, non-coding RNAs

## Abstract

Cardiovascular diseases (CVDs) caused by arteriosclerosis are the leading cause of death and disability worldwide. In the late stages of atherosclerosis, the atherosclerotic plaque gradually expands in the blood vessels, resulting in vascular stenosis. When the unstable plaque ruptures and falls off, it blocks the vessel causing vascular thrombosis, leading to strokes, myocardial infarctions, and a series of other serious diseases that endanger people's lives. Therefore, regulating plaque stability is the main means used to address the high mortality associated with CVDs. The progression of the atherosclerotic plaque is a complex integration of vascular cell apoptosis, lipid metabolism disorders, inflammatory cell infiltration, vascular smooth muscle cell migration, and neovascular infiltration. More recently, emerging evidence has demonstrated that non-coding RNAs (ncRNAs) play a significant role in regulating the pathophysiological process of atherosclerotic plaque formation by affecting the biological functions of the vasculature and its associated cells. The purpose of this paper is to comprehensively review the regulatory mechanisms involved in the susceptibility of atherosclerotic plaque rupture, discuss the limitations of current approaches to treat plaque instability, and highlight the potential clinical value of ncRNAs as novel diagnostic biomarkers and potential therapeutic strategies to improve plaque stability and reduce the risk of major cardiovascular events.

## Introduction

Atherosclerosis (AS) is a chronic and complex pathological process that is the major cause of cardiovascular diseases. Genetic susceptibility and environmental factors (*e.g.* smoking, hypertension, hyperlipidemia, diabetes, family history, and obesity) are the main factors that influence AS, with plaque accumulation in the arterial wall being one of the most prominent features 1. A stable plaque becomes unstable and vulnerable to rupture as AS progresses. Factors that negatively affect plaque stability include necrosis in the plaque core, the presence of inflammatory cells, and thinning of the fibrous cap structure 2, 3. Moreover, neovascularization in unstable plaques is more likely to exacerbate the rupture process compared to that in stable plaques 4. While an increase in vessel permeability is conducive to the infiltration of inflammatory cells, the increase in vessel fragility results in easy bleeding and the formation of hematomas in plaques. In the late stages of atherosclerosis, the thin and fragile fiber cap with a large necrotic core, a large number of infiltrated inflammatory cells, intra plaque hematomas, and secondary thrombosis all aggravate the development of AS resulting in serious cardiovascular diseases such as coronary artery disease (CAD), acute coronary syndrome (ACS), myocardial infarction, and stroke 1, 5, 6. Therefore, maintaining plaque stability and preventing plaque rupture are widely considered to be the principle goals in the clinical treatment of patients with cardiovascular diseases 7.

As discussed above, atherosclerotic plaques are divided into stable plaques and unstable plaques. A stable plaque is characterized by a small swollen and a thick fibrous cap 8; there is also an absence of clinical symptoms. Conversely, the characteristics of the rupture-prone plaques are (a) a thin fibrous cap with a large lipid core; (b) infiltration of monocytes and macrophages; (c) aggregation of endothelial exfoliation and the presence of surface platelets; (d) deposition of extracellular dense lipid droplets and formation of large lipid droplets around the nucleus; and (e) fibrous connective tissue that forms part of the plaque 9. The formation of ulcers or fissures in unstable plaques can release or activate transmitters, such as thrombin, adenosine diphosphate (ADP), platelet activating factor (PAF), tissue factor, and oxygen free radicals, which promote platelet aggregation and aggravate arterial mechanical obstruction. Plaque instability is therefore an important factor affecting the occurrence and development of AS, but the mechanisms leading to the instability of atherosclerotic plaques remain to be determined.

Recent studies have shown that non-coding RNAs (ncRNAs) are critically involved in the regulation of plaque instability 10-12. NcRNAs can not only participate in regulating the expression of lipoproteins and impact the proliferation and differentiation of vascular smooth muscle cells (VSMCs) to affect plaque stability but also regulate the phenotypic transition of immune cells to affect the development of inflammation around plaques. Encouragingly, several microRNAs (miRNAs), long non-coding RNAs (lncRNAs) and small interfering RNAs (siRNAs) have been found to be differentially expressed in the serum from patients with AS and have shown remarkable capabilities in regulating plaque stability, indicating their potential to be used as diagnostic biomarkers or as therapeutic targets 13-18.

Studies examining the relationship between plaque stability and extracellular vesicles and exosomes have also made great progress. For example, it has been shown that the accumulation of extracellular vesicles can aggravate plaque calcification and promote a vasoactive response 19, 20; changes in calcification morphology and collagen content in plaques are also associated with exosomes 21. Furthermore, exosomes are thought to be promising carriers for nuclear drugs 22. Several studies have also presented evidence that exosome delivery systems can control plaque instability 23.

In this review, we introduce the cellular and molecular mechanisms of atherosclerotic plaque stability and highlight the role of ncRNAs in plaque vulnerability. We also summarize new progress in the use of exosomes to treat plaque instability. Finally, we discuss the limitations of the current studies and provide emerging insights into the role of ncRNAs in the regulation of atherosclerotic plaque stability and their potential as targets for novel therapeutic paradigms.

## Cells involved in the regulation of plaque instability

### Endothelial cells

The endothelium plays a pivotal role in the progression of AS and its complications, and endothelial dysfunction is widely recognized as one of the early alterations in the vessel wall preceding the development of plaques 24, 25. Emerging evidence has shown that the degree of endothelial cell (EC) apoptosis may be a key factor in the transition of a plaque from a stable state to a fragile state 26, 27. The glycolytic enzyme 6-phosphofructo-2-kinase/fructose-2,6-biphosphatase (PFKFB3) is highly expressed in the ECs present in vulnerable human carotid atherosclerotic plaques, and inhibition of PFKFB3 activity reduces cell apoptosis in plaques and promotes plaque stability 28. Knockout of the Dickkopf1 (*DKK1*) gene in apolipoprotein E-deficient (ApoE^-/-^) mice inhibits the classical Wingless-Related Integration Site (WNT) signal by activating the JNK1 signal transduction pathway and reduces the vulnerability for apoptosis in human umbilical vein endothelial cell (HUVEC) in the presence of oxidized low density lipoprotein (ox-LDL) during the AS process 29. Moreover, the apoptosis of ECs also leads to local lipid deposition in blood vessels 30, aggravates plaque rupture, and may predispose individuals to arterial thrombosis. In addition, the migration of ECs and the increase in vascular permeability are closely related to the progression and instability of atherosclerotic plaques. Vascular endothelial growth factor receptor 2 (VEGFR2) can induce the expression of disintegrin and metalloprotease 10 (ADAM10) in ECs, and the combination of VEGFR2 and ADAM10 can promote the migration of ECs and accelerate the progression of atherosclerotic plaques 31. Furthermore, the endothelial to mesenchymal transition (EndMT) plays a key role in cardiovascular disease and the rupture of unstable plaques because EndMT-derived cells are found in oxidative stress and hypoxia-induced atherosclerotic plaques 32. Chen et al*.* found that in 25-week-old ApoE^-/-^ mice fed a standard diet, an EC-specific nucleotide P2Y2 receptor (P2Y2R) deficiency prevented vascular adhesion molecule-1 (VCAM-1) in ECs from participating in vascular inflammation and reduced nitric oxide (NO) synthase and matrix metalloproteinase-2 (MMP-2) activity owing to a decrease in macrophage infiltration 33. A recent intriguing finding has described the protective effect of oleic acid on cardiovascular cells through an inhibition of tumor necrosis factor-α (TNF-α) and a decrease in the expression levels of monocyte chemoattractant protein-1 (MCP-1) and ICAM-1, thereby improving endothelial dysfunction 34, 35. In general, the key factor affecting the stability of atherosclerotic plaques is endothelial dysfunction, which is mainly manifested by impaired endothelial barrier function, increased vascular permeability, and EC apoptosis (Fig. 2).

### Vascular smooth muscle cells

The majority of VSMCs in plaques are derived from the medial layer of the blood vessel. The plasticity of VSMCs play an important role in the occurrence and development of AS and can protect the fibrous cap from rupture and participate in the synthesis of extracellular matrix components 36. VSMC phenotypic remodeling plays an important role in the early stage of AS 37, 38. Under pathological inflammatory conditions, VSMCs initially with a stable and normal vasoconstriction phenotype undergo remodeling to a synthetic phenotype. In this latter state, VSMCs express immunoregulatory cytokines, such as IL-6 and CC chemokine ligand (CCL)2, and secrete chemokines to activate the inflammatory state of macrophages, thereby affecting the local stability of the plaques 39, 40. Serum response factor (SRF), as a key regulator of vascular inflammation, is an important player that regulates the phenotype of VSMCs. Increased expression of SRF reduced the accumulation of macrophages in ApoE^-/-^ mice and inhibited VSMC phenotype changes and the activation of inflammation, thereby enhancing plaque stability 41, 42. Conditional knockout of the *KLF4* gene has been shown to reduce the number of VSMC-derived macrophages and mesenchymal stem cells, resulting in an increase in fibrous cap thickness and a decrease in lesion size 43. Therefore, VSMC contributes to the stability of atherosclerotic plaques through a KLF4-dependent phenotypic transformation mechanism. In keeping with this, it has also been shown that deletion of AMPKα2 promotes the phenotypic conversion of contractile VSMCs to synthetic VSMCs by increasing KLF4 expression 44. VSMCs are the main source of collagen in the fiber cap (FC) which is responsible for its tensile strength. A reduction in the number of VSMCs due to the death of initiating cells leads to FC thinning, necrotic nucleus formation, and calcification 1. Additionally, the Fas receptor/Fas ligand pathway is involved in ox-LDL-induced apoptosis of VSMCs. Interestingly, the activation of p53 makes the VSMCs more sensitive to Fas-mediated apoptosis by transiently increasing Fas expression and translocation from the Golgi 45, 46. In addition, DNA damage in VSMCs has been shown to be involved in human atherosclerotic plaques both *in vitro* and *in vivo* 47, 48, which are manifested by double strand breaks (DSBs) and the increased expression of multiple DNA damage response proteins. Among these, the nuclear deacetylase sirtuin6 (SIRT6) has been reported to participate in the DNA damage response. The overexpression of SIRT6 in VSMCs reduced the activity of nuclear factor-kappa B (NF-κB)-dependent inflammatory factors, inhibited cell senescence, and protected atherosclerotic plaques in ApoE^-/-^ mice 49. Therefore, inhibiting DNA damage in VSMCs by reducing the relative FC area of late-stage plaques could be a promising target for creating clinically stable plaques 50. The decreased activity of MMP-2, and the increased migration ability of VSMCs, can increase plaque stability 51. Early aging of VSMCs and an increased sensitivity to apoptosis in atherosclerotic plaques reduces the ability to repair vulnerable plaques. In addition, the abnormal proliferation of VSMCs after phenotypic transformation also accelerates the process of plaque rupture. Therefore, identifying the factors and mechanisms that can promote the phenotypic transformation of VSMCs and improve plaque stability is an important goal to prevent plaque rupture in the future (Fig. 3).

### Immune cells

In the development of AS, phenotypic changes and cytokines secreted by immune cells such as monocytes, macrophages, dendritic cells, and mast cells can stimulate inflammation and affect the stability of plaques. Therefore, understanding the mechanisms by which immune cells promote inflammation and how changes in cell function arise owing to alterations in immune cell surface receptors is likely to be very important in learning how to stabilize plaques and ameliorate the progression of AS.

### Monocytes and Macrophages

Chemokines mobilize monocytes to migrate and adhere to ECs, which is a key process in macrophage aggregation 52. Interestingly, the Src family-associated kinases HCK and FGR, mediate the interaction between EC adhesion molecules and β-2 integrins and participate in a series of AS-related processes through activation of Rac/Cdc42, Syk, and Pyk7 effectors. They also lead to dynamic instability of the extracellular matrix by causing an imbalance in monocyte subsets, making the plaque more prone to rupture 53. Monocyte to macrophage differentiation plays an important role in the early stages of AS 54, and the accumulation of macrophage-derived foam cells in the arterial wall promotes monocyte adhesion and infiltration 55, 56. Ma et al*.* found that formononetin can inhibit monocyte adhesion and enhance plaque stability by reducing the expression of SRA in monocytes 57. Insulin-like growth factor-1 (IGF-1) is highly expressed in monocytes/macrophages in ApoE^-/-^ mice. IGF1R-deficient macrophages inhibit the expression of ABCA1 and ABCG1 and reduce lipid efflux and plaque vulnerability 58. Interestingly, the activation of MC1-R can not only prevent macrophages from accumulating lipid but also promote the reverse transport of cholesterol by up-regulating the levels of ABCA1 and ABCG1 and reducing the expression of CD36 on the cell surface 59, 60. Li et al. found that overexpression of C1q/TNF-related protein 9 (CTRP9) can reduce the levels of pro-inflammatory factors such as TNF-α and MCP-1 in macrophages 61. Wen et al*.* showed that the levels of phosphorylated ERK-MAPK, p38-MAPK, and JNK-MAPK were significantly reduced by pigment epithelium-derived factor (PEDF) through the regulation of the PPAR-γ and downstream MAPK inflammatory pathways, so as to reduce macrophage inflammation and increase plaque stability 62. Additionally, several studies have shown that late plaques contain more apoptotic cells than early plaques 58, 63, and the occurrence of modified LDL in the process of early plaque efferocytosis can induce macrophage apoptosis. However, defects will occur with the further development of plaque efferocytosis, resulting in plaque rupture and remodeling of the plaque structure 64. Therefore, macrophage apoptosis is another key factor in plaque instability (Fig. 4).

### Dendritic cells

The high cholesterol environment in patients with atherosclerosis leads to a decrease in the migration of DCs because of the engulfment of the walls of blood vessels with their own antigens, such as ox-LDL. The number of tolerogenic DCs increased when ox-LDL -induced apoptotic DCs (apop^ox^-DCs) were injected intravenously into LDL^-/-^ mice. Interestingly, the collagen levels in mice treated with apop^ox^-DCs were increased by 45% compared with untreated apoptotic dendritic cells, significantly reducing the progression of lesions, which is suggestive of increased plaque stability 65. In vulnerable plaques, the number of mature DCs is significantly increased and these mature DCs play an important role in the inflammatory processes in atherosclerotic lesions by stimulating effector T cells. Because of the interaction between DCs and regulatory T cells (Treg), there is a direct inhibition of DC migration and adhesion to ECs, leading to the development of plaque instability 66. In atherosclerotic tissues, the myeloid cell receptor referred to as triggering receptor in myeloid cells (TREM)-1, is a key factor in inflammation, and that the number of DCs increases gradually with the progression of the disease. The expression of TREM-1 in DCs is significantly increased in plaques of patients with symptomatic carotid stenosis, indicating that both DCs and TREM-1 may play an important role in plaque stability 67.

### Mast cells

Mast cells are a type of multifunctional white blood cells, which are mainly found in mucosal tissues and connective tissues. An abnormal increase in mast cell number is often accompanied by the occurrence of cardiovascular disease. It is thought that several mast cells and their resultant activation affects the stability of plaques. To further understand the role of the vascular network as a transport channel for several angiogenic and plaque forming factors, Joosp et al*.* Used indocyanine green video angiography (ICG-VA) during a carotid endarterectomy (CEA) to investigate the correlation between the change in state of the carotid artery and the vulnerability of a carotid plaque and showed that mast cells stained with CD117 were more frequently found in unstable plaques than in stable ones 68. Mast cells are activated by the binding and cross-linking of antigens to IgE which is itself bound to the Fcε-receptor (FcεR). This results in the release of cytoplasmic granules which contain proinflammatory factors, histamine, and several neutral proteinases. Kritikou et al. used improved flow cytometry to identify specific mast cells that expressed both CD117 and FcεR at the same time and observed that the activation of mast cells in most plaques depends on the expression of the CD63 protein and the presence of IgE fragments on their surfaces 69. This strongly suggests that the development of AS is related to the number of mast cells and the activation of their surface receptors and, therefore, provides a new strategy for the clinical treatment of AS.

### Lymphocytes

At present, while there is no direct research that supports the involvement of lymphocytes in the formation and development of plaques, the possibility has been suggested. For example, Tsaousi et al*.* suggested that knockout of the T-bet gene inhibits Th1 lymphocyte differentiation to reduce the expression of the M1 macrophage marker NOS-2 and, consequently, the size of atherosclerotic plaques 70. These phenomena suggest that lymphocytes may be associated with plaque instability, but this needs to be further explored.

## Mechanisms by which plaque instability is regulated

### Inflammation

Inflammation is widely considered to play a critical role in the formation of atherosclerotic plaques and plaque rupture 71-74. Histopathology of lateSvulnerable plaques has shown that there are obvious signs of inflammation. In addition, the action of excessive proteolytic enzymes, which stimulate macrophages to participate in the immune response, inhibits the formation of the fibrous cap and degrades the fiber composition of the cap 5. The accumulation of activated macrophages, T cells, and necrotic core lipids in vulnerable plaques triggers a self-persistent, vicious cycle of inflammatory responses 75, 76. In patients with AS, activated NLRP3 inflammasomes produce IL-1β and IL-18 77, 78, which up-regulate VCAM to induce T-cell differentiation and promote a downstream inflammatory response, thus leading to plaque progression 79, 80. Interferon-γ, a pro-inflammatory cytokine produced by Th1 T cells and natural killer (NK) cells, makes plaques more vulnerable by inhibiting smooth muscle cell differentiation 81 and interstitial collagen gene expression 82. Likewise, the production of TGF-β can inhibit the activity of Th1 cells and macrophages and reduce plaque inflammation 25, 83. The cytokine IL-17A alleviates the deleterious mechanical effects of hemodynamics on plaques by promoting collagen gene expression 84, 85. In addition, the plaque expression levels of the pro-inflammatory factor leukotriene B (LTB) (4) are up-regulated by the BLT1 receptor, which could be a target for treating plaque instability 86. Leukotriene receptors and their important cofactor 5-lipoxygenase-activating protein (FLAP) are highly expressed in atherosclerotic plaques and promote the production of LTB4 87. The levels of the formyl peptide receptor (FPR) subtype FPR2/ALX are significantly increased in atherosclerotic lesions. FPR2/ALX has pro-inflammatory and plaque destabilizing effects on myeloid-derived cells but has the opposite effect on VSMCs 88. The tyrosine kinase inhibitor AG1296 which inhibits the inflammatory response by blocking the platelet-derived growth factor/platelet-derived growth factor receptor (PDGF/PDGFR) signaling pathway can reduce the expression levels of MMP-2 and MMP-9 to enhance plaque stability 88, 89. In recent years, the Colchicine Cardiovascular Outcomes (COLCOT) and the Canakinumab Anti-inflammatory Thrombosis Outcomes Study (CANTOS) trials targeting NLRP3/IL-1β pathway have provided potential evidence to treat AS 90, but it is still unknown whether promoting the immune response in patients with AS affects the normal immune homeostasis. Therefore, how to specifically identify vulnerable plaques and target the immune response in the lesions of patients with AS is the next problem that has to be solved (Fig. 4).

### Lipid metabolism

Another main reason for the development of AS is the elevated levels of LDL, leading to cholesterol accumulation in the intima, which eventually attracts monocytes. Macrophage phagocytosis of ox-LDL resulting in the creation of foam cells can accelerate the progression of AS 91. Rinne et al. found that activation of MC1-R in macrophages in atherosclerotic ApoE^-/-^ mice increased the outflow of cholesterol to that of ApoA1 and high-density lipoprotein (HDL), because of the increased reverse transport of cholesterol mediated by ATP-binding cassette transporters ABCA1 and ABCG1 to make the plaque stability signs more obvious 92. There is evidence that there is an increase in protein deacetylation in macrophages from ApoE^-/-^ mice treated with hydrogen sulfide (H_2_S), leading to increased deacetylation of several proteins including p53, p65, and sterol response element binding protein. As a result, there is an inhibition of both the synthesis of liver cholesterol and the uptake of cholesterol by macrophages, thereby increasing plaque stability and reducing plaque formation 93. ApoA-I is the main structural protein component of HDL particles and has been shown to play a key role in reverse cholesterol transport (RCT) 94; thus, stimulating an increase in endogenous ApoA-I synthesis may be a promising treatment for plaque instability.

### Oxidative stress

The excessive production of reactive oxygen species (ROS) is another reason for the development of AS and the formation of unstable plaques. Evidence supports that a dysfunction in endothelial NO synthase (NOS) and an up-regulation of vascular NADPH oxidase in AS are closely related to plaque instability 95. Ox-LDL activates NOS1 and leads to the expression of CD40 ligand in macrophages. Inhibition of NOS1-derived NO may therefore be an effective strategy to reduce foam cell formation and limit the extent of atherosclerotic plaque expansion 96. There is evidence that NOX2, a specific inhibitor of NADPH oxidase, can inhibit superoxide dismutase and significantly reduce the expression of hypoxia-inducible factor-1 α, MMP-9, endothelin, and vascular endothelial growth factor in a ApoE^-/-^ mouse model fed a high-fat diet. Therefore, NOX2 can stabilize atherosclerotic plaques and reverse atherosclerotic vascular lesions 97. In addition, the loss of the transcriptional activator Arnt-like protein-1 (BMAL1) in human aortic endothelium inhibits the intracellular ROS accumulation induced by ox-LDL through BMP-mediated signal transduction, which further aggravates EndMT and negatively affects plaque stability 98. Deletion of the transcription factor NF-E2 related factor 2 (Nrf2) reduced the extent of atherosclerotic lesion formation in LDLR^-/-^ mice but also led to an increase in the plaque instability index by increasing plaque calcification and oxidative stress in 12-month-old LDLR-/-ApoB100/100 female mice 99-101. It has further been confirmed that Nrf2 plays a completely different role in different animal models as well as in early and late plaques. In addition, in mouse models of AS, it is clear that there is gender dimorphism in the development of AS. These findings have provided us with new ideas for development of drugs to treat plaque instability in the future.

### Exercise training

In patients with stable coronary heart disease (CHD), high degrees of physical activity are associated with low mortality 102, 103. Additionally, exercise training has been shown to prevent AS and angiotensin (Ang)-induced vulnerable plaque formation in ApoE^-/-^ mice by reducing systemic inflammation 104. Moderate aerobic exercise can convert calcium deposits in blood vessels into a more stable form 105 and also stabilize plaques by increasing their collagen content 106, 107. More interestingly, recent studies have shown that exercise may reduce macrophage activity by down-regulating neuropeptide Y (NPY) receptor expression in ApoE^-/-^ mice and increase plaque collagen levels and smooth muscle cell numbers that play a stabilizing role in plaques 108.

## Vital roles of ncRNAs in plaque instability

Ample studies are available demonstrating the association between ncRNAs and plaque stability, which provide a rationale for the development of ncRNA-targeted therapeutic strategies in AS (Table 1).

### ncRNAs in ECs

The effects of endothelial dysfunction, inflammation, ROS, and NO production on plaque stability are obvious. The blood flow in atherosclerotic lesions is characterized by turbulent dynamics, and these hemodynamics have a far-reaching impact on the expression of miRNAs, with differentially regulated miRNAs modulating shear stress-mediated transcriptional procedures 109-111. It has been found that the levels of miR-200C are significantly higher in unstable plaques than in stable plaques. There is also a positive correlation between miR-200C levels and markers of plaque instability. Surprisingly, blood miR-200C expression levels decreased in patients with stable plaques one month after CEA. In addition, it has been found that H_2_S regulates the stability of NO synthase in ECs by up-regulating miR-455-3p to promote the migration of HUVECs. These data suggest that miR-455-3p is closely related to plaque instability and atherosclerotic progression 112. Recent studies have shown that hsa_circ_0030042 can ameliorated plaque stability and regulates abnormal autophagy by targeting eukaryotic initiation factor 4A-III (eIF4A3) 113. The overexpression of miR-19b can inhibit the transcriptional activity of the transcriptional activator 3 (STAT3) 114. Another study has shown that decreased lncRNAUC.98 expression can stabilize the progression of atherosclerotic plaque by inhibiting the proliferation and migration of ECs 115. A reduction in miRNA-27b levels has been shown to regulate the activity of the chemokine (C-C motif) ligand 20/C-C chemokine receptor type 6 (CCL20/CCR6) axis by targeting N-alpha-acetyltransferase 15 (Naa15) to promote the stability of atherosclerotic plaques 116. LncRNA AK136714 silencing inhibits endothelial cell inflammation and protects plaque stability 117. The lncRNA TCONS_00024652 acts as miR-21 sponge to regulate vascular endothelial cell proliferation and angiogenesis and may be used as a potential method to reduce vascular endothelial dysfunction and treat plaque instability 118.

### ncRNAs in VSMCs

The increase in plaque size and decrease in plaque stability caused by the transformation of VSMCs into foam cells is a key step in the formation of AS plaques 43. Gabunia K et al*.* reported that interleukin-19 (IL-19), a new type of anti-inflammatory cytokine, has been shown to reduce lipid accumulation in VSMCs. MiR-133a can target and decrease the mRNA levels, stability, and protein expression levels of the LDL receptor adapter protein 1 (LDLRAP1). Mutations in miR-133a lead to LDL receptor dysfunction resulting in human autosomal recessive hypercholesterolemia (ARH). Therefore, miR-133a is a new target to reduce plaque size and rupture vulnerability by reducing Ox-LDL uptake in VSMCs 119. High expression levels of miR-145 in VSMCs have recently been found to regulate AS and plaque stability. Targeting miR-145 in VSMCs has been shown to increase the area of the fiber cap, collagen content, and plaque stability 120. Contrary to the effects of miR-145, miR-124-3p inhibits VSMC collagen synthesis by directly targeting prolyl 4-hydroxylase subunit alpha-1 (P4HA1), resulting in atherosclerotic plaque instability 121. MiR-210 enhances the stability of the fibrous cap in advanced atherosclerotic lesions by targeting the tumor suppressor gene adenomatous polyposis coli (*APC*), thereby affecting WNT signal transduction, regulating VSMC apoptosis, and preventing plaque rupture 122. On the one hand, miR-21 can affect the formation of foam cells and the development of local lipid cores by regulating the activity of the NF-κB signal pathway. On the other hand, miR-21 can inhibit VSMC apoptosis by increasing the rate of proliferation of VSMCs in mouse carotid arteries and jointly protecting fibrous cap stability in atherosclerotic plaques 123. It has been shown that hyperglycemia and streptozotocin-induced type 1 diabetes can reduce the synthesis of collagen and lead to the formation of unstable atherosclerotic plaques in ApoE^-/-^ mice 124. Metformin can increase the level of activator protein 2 alpha (AP-2α) in carotid atherosclerotic plaques in diabetic ApoE^-/-^ mice, decrease the expression levels of miR-124, and increase the levels of prolyl-4-hydroxylase alpha 1 (P4Hα1) and collagen in VSMCs. Therefore, targeting miR-124 can increase plaque stability by regulating the activity of the AMPKα/AP-2α/miRNA-124/P4Hα1 axis, which provides a new idea for the clinical treatment of AS 125.

### ncRNAs in macrophages/monocytes

**Figure 1:**
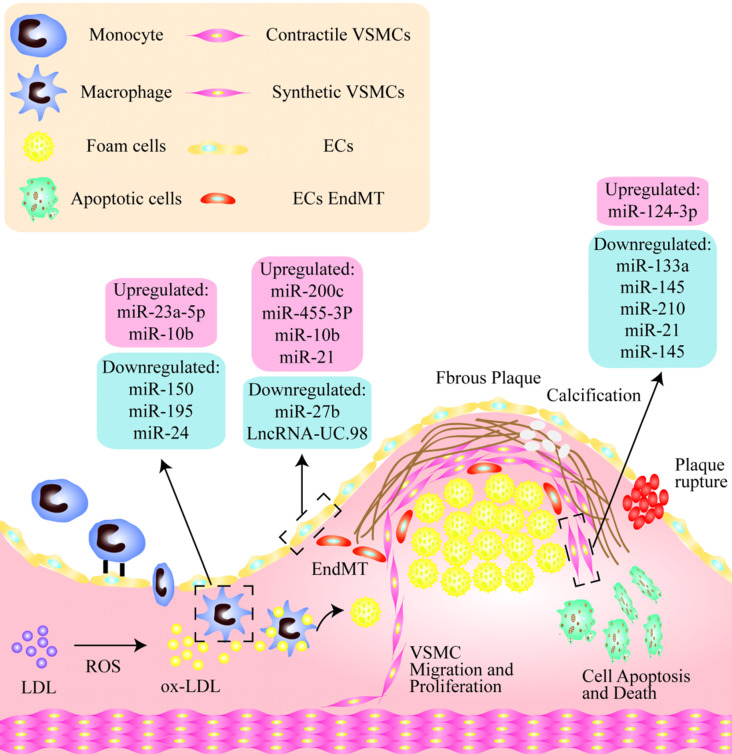
** Non-coding nucleic acids participate the regulation of unstable plaques via mediating functions of endothelial cells, vascular smooth muscle cells and macrophages.** As shown, there is a thin fibrous cap in the unstable plaque, and the vulnerable sites are rich in lipid cores, including a large number of apoptotic cells, cholesterol crystals, lipid-rich foam cells, and so on. Among them, in endothelial cells, miR-200C, miR-455-3p, miR-10b and miR-21 were significantly up-regulated, while miR-2b and LncRNA-UC.98 were down-regulated. miR-124-3p was increased and miR-133a, miR-145, miR-210, miR-21, miR-145 were significantly decreased in VSMCs. In addition, miR-23a-5p and miR-10b were up-regulated in macrophages, while miR-150, miR-196 and miR-24 were obviously down-regulated.

**Figure 2:**
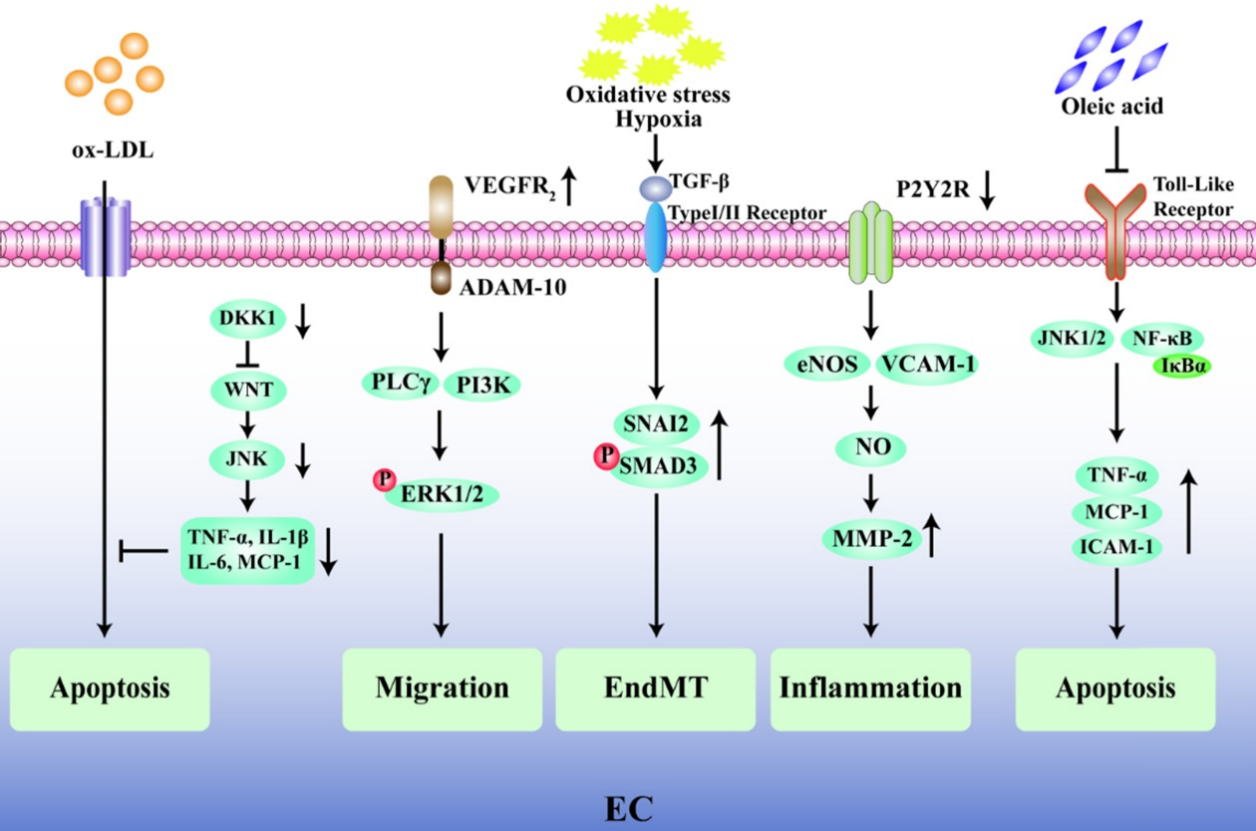
** Regulatory mechanism of endothelial cells in unstable plaques.** Inhibition of endothelial cell apoptosis induced by ox-LDL was mainly through WNT/JNK pathway after Dickkopf1 (*DKK1*) silencing. The combination of vascular endothelial growth factor receptor 2 (VEGFR2) and its ligand metalloprotease 10 (ADAM10) induces the phosphorylation of ERK1/2 through PLCγ/PI3K signal transduction and promotes the migration of ECs. Oxidative stress and hypoxia induce transforming growth factor-β (TGF-β) to stimulate the phosphorylation of SNAI2 and SMAD3, resulting in endothelial to mesenchymal transition (EndMT). P2Y2 receptor (P2Y2R) deletion can promote the expression of vascular adhesion molecule-1 (VCAM-1) and endothelial nitric oxide synthase (eNOS) in ECs, thus promote the activity of nitric oxide (NO) and matrix metalloproteinase-2 (MMP-2), then increase endothelial inflammation. Oleic acid suppresses cell apoptosis by inhibiting Toll-like receptor-activated JNK/NF-κB/IκBα pathway and the expression of TNF-α, MCP-1 and ICAM-1.

**Figure 3:**
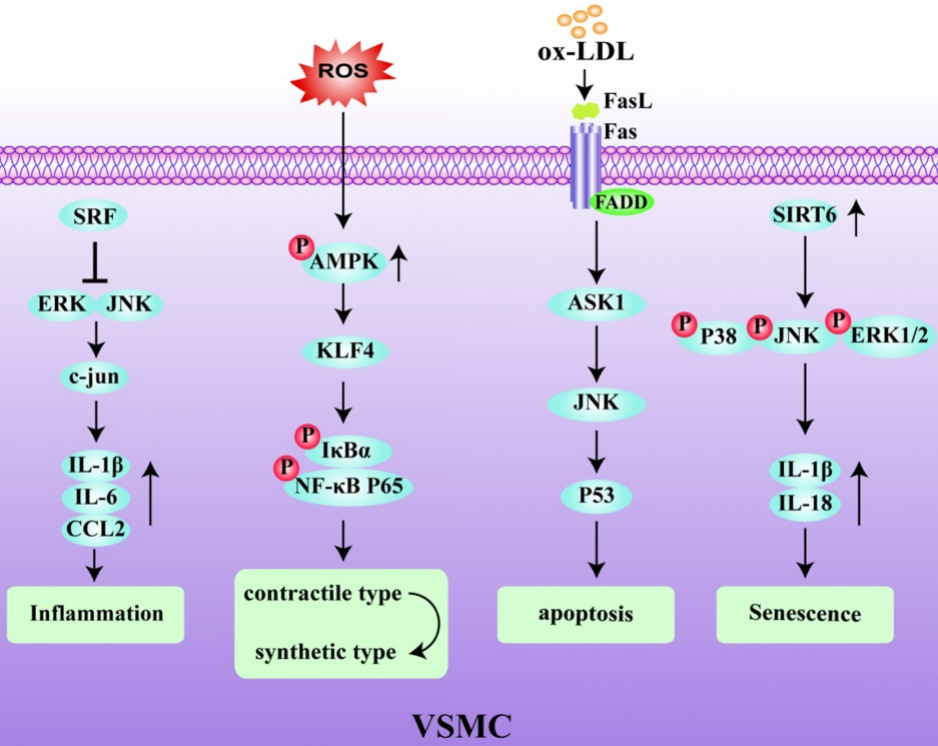
** Regulatory mechanism of VSMC in unstable plaques.** Serum response factor (SRF) blocks the inflammatory pathway by inhibiting ERK/JNK/C-Jun pathway and the expression of IL-1β, IL-6 and CCL2. ROS activates the phosphorylation of AMPK and induces phenotypic transformation of VSMCs through the klf4-dependent IκBα/NF-κB p65 pathway. Sirtuin6 (SIRT6) promotes the increase of IL-1β and IL-18 and induces VSMCs senescence by activating the phosphorylated p38/JNK/ERK1-Beat 2 pathway.

**Figure 4:**
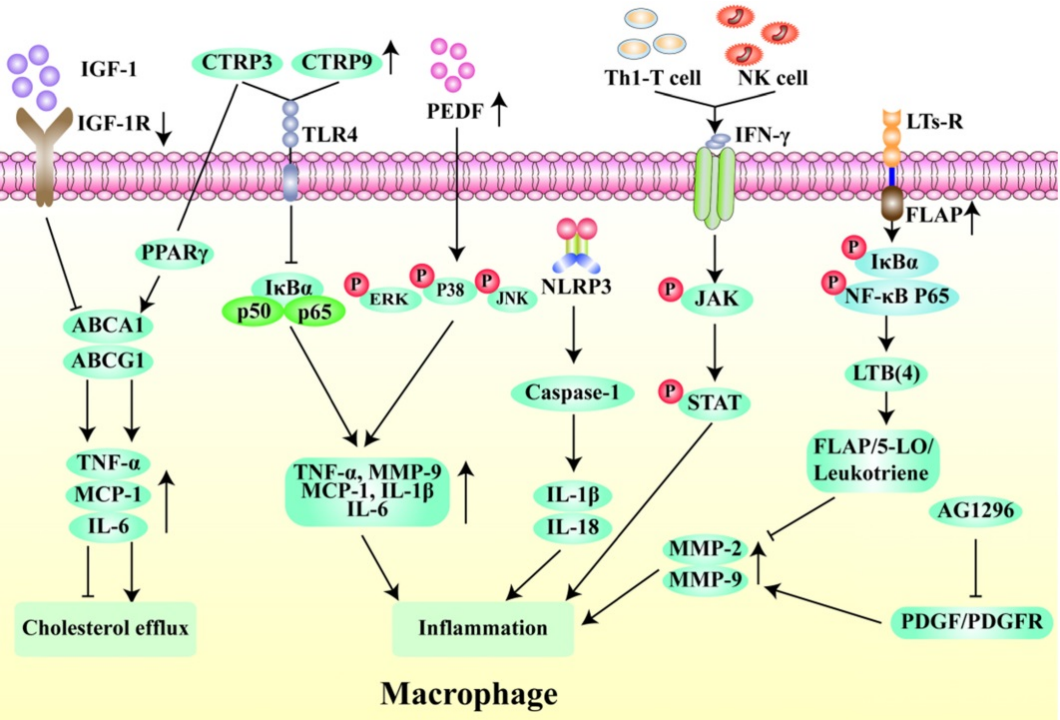
** Regulatory mechanism of macrophages in unstable plaques.** Insulin-like growth factor-1 receptor (IGF-1R) deletion inhibits cholesterol outflow by suppressing the expression of TNF-α, MCP-1 and IL-6 through ABCA1/ABCG1 pathway. In contrast, C1q/tumor necrosis factor-related protein-3 (CTRP3) promotes cholesterol outflow through PPAR-γ. The high expression of CTRP3 and CTRP9 can inhibit the macrophage inflammation through NFκB pathway. Pigment epithelium-derived factor (PEDF) can activate ERK, p38 and JNK phosphorylation to inhibit macrophage inflammation. NLRP3 inflammasomes induces inflammation by activating IL-1β and IL-18. Interferon-γ produced by Th1 T cells and natural killer (NK) cells activates the phosphorylation of JAK and STAT to promote inflammation. Leukotriene receptors (LTs-R) and its cofactor FLAP target FLAP/5-LO/Leukotriene pathway to inhibit inflammation. The tyrosine kinase inhibitor AG1296 suppresses inflammation by reducing the expression of MMP-2 and MMP-9 via PDGF/PDGFR pathway.

NcRNAs are critically involved in macrophage apoptosis, inflammation, and the phenotypic transformation leading to plaque instability. It has been shown that the serum levels of miR-23a-5p in patients with AS as well as in macrophages in atherosclerotic mice are both significantly increased. A miR-23a-5p inhibitor has been shown to increase cholesterol efflux and reduce the formation of foam cells by up-regulating the expression of ABCA1/G1. A miR-23a-5p anticoagulant therapy has been shown to significantly slow the progression of AS probably by inhibiting an ATP-binding cassette transporter in macrophages that promotes the progression and vulnerability of atherosclerotic plaques 114. Analogously, the expression levels of miR-10b are increased in the arteries of late atherosclerotic plaques in ApoE^-/-^mice 126. In addition, free cholesterol-induced macrophage apoptosis (FC-AM) has been shown to promote the expression of miR-10b in resident peritoneal macrophages (RPM) by up-regulating Mer receptor tyrosine kinase-dependent Twist1/2 127. It has been shown that miR-150 significantly enhances the inflammatory response by up-regulating the proliferation, migration, and vascular homeostasis of ECs. It can also reduce infiltration and lipid accumulation in macrophages to promote plaque stabilization 128. Some studies have found that miR-195 participates in the polarization of macrophages and inhibits mediators of the Toll-like receptor 2 (TLR2) inflammatory pathway. In addition, miR-195 weakens the effect of macrophages on the recruitment and migration of VSMCs 129. Recent studies have suggested that membrane-1 MMP-14, a selective marker of a subset of invasive macrophages, has been shown to be associated with atherosclerotic plaque progression. The levels of miR-24 in stable plaques are higher than in unstable plaques, and the downregulation of miR-24 promotes the formation of the subset of invasive macrophages, suggesting a new regulatory role for MMP-14 proteolytic activity in plaque stability 130 (Fig. 1).

## Clinical applications

The rupture of unstable atherosclerotic plaques and thrombosis are the most important pathological basis of AS which seriously threatens the life of patients. Therefore, early diagnosis and the identification of unstable plaques are of great value to patients with coronary heart disease 131-134.

Clinically, intravascular ultrasound (IVUS) and optical coherence tomography (OCT) can provide the morphological characteristics of coronary atherosclerotic plaques, which is very helpful for the assessment of plaque stability 135-137. However, these tools are too invasive and expensive to be widely used in the screening of unstable plaques. Strategies targeting circulating biomarkers, such as ncRNAs, could provide a more convenient method to evaluate plaque stability in patients with coronary heart disease 15, 138, 139. There are several advantages for ncRNA application in clinical trials. RNA therapy can specially mediate target genes. NcRNAs are associated with complex biological processes such as immune cell development and functions 140-142. The preclinical development of ncRNA drugs is simpleness in design through gene sequencing. Moreover, the treatment of small nucleic acid drugs at the post-transcriptional level can suppress the activity of special targets whose proteins are difficult to be effective. Besides, RNA therapy can also mediate multiple targets at the same time 143. For example, a biomimetic exocrine nanocomplex for accurate delivery of miRNA has been developed, which shows excellent targeted delivery and therapeutic effect in mouse myocardial infarction model, which provides a novel idea for the design and development of nucleic acid drug carriers, realizes the accurate delivery of gene drug and microRNA-mediated myocardial repair, and provides a theoretical basis for nucleic acid therapy 144. Therefore, targeting ncRNAs would be a very promising strategy for the treatment of diseases in future. Panz et al*.* sequenced the transcripts of blood samples from three patients with stable plaques and three patients with unstable plaques by RNA sequencing, and found 62 species of lncRNAs were differentially expressed in unstable plaques. In particular, lncRNA-snhg7-003 was found to be significantly down-regulated in blood samples from patients with unstable plaques 145. A microarray analysis of plasma from five patients with AS showed that the expression levels of miR-23a-5p, miR-2110, and miR-320a were all up-regulated compared with those in plasma from healthy controls. In parallel, the levels of miR-4439 and miR-8084 were down-regulated in plasma from patients with vulnerable plaques 114. The miRNA expression profile of human atherosclerotic plaques has been analyzed using a miRNA chip. It has been found that the expression levels of miR-21, miR-22, miR-210, miR-34a, miR-146a/b, miR-19b, and miR-143 were all clearly up-regulated 146, 147. Moreover, miR-99b, miR-152, and miR-422a have also been shown to be highly expressed in atherosclerotic plaques but not in healthy blood vessels 148. Li et al. identified miR-27b as having the higher expression levels in atherosclerotic plaques from high-fat diet-fed ApoE^-/-^mice 116. In addition, miR-145 has been shown to be overexpressed in plaques in patients with hypertension 149. High levels of miR-100 have been shown to be associated with coronary plaques 150, and carotid plaque rupture is accompanied by an increase in the serum circR-284/miR-221 ratio 151. Therefore, ncRNAs may represent potential diagnostic markers to regulate plaque stability. In the context of human atherosclerotic diseases, the precedent of targeted miRNA therapy has been confirmed. So far, it has been shown that knocking down the expression of miR-27b, miR-210, miR-520c-3p, and lncR-*ccl2* protects plaque stability in ApoE^-/-^ mice 116, 122, 152, 153; therefore, these attractive targets have a huge potential to regulate plaque stability. Regarding plaque stability, the plaques in the widely used ApoE^-/-^ or LDLR^-/-^ mice are usually less prone to rupture as compared to atherosclerotic plaques in humans. Over the past decade, ncRNAs have become the main pathophysiological regulators of atherosclerosis, but most of these studies are based on mice, and conducting extensive studies in humans is an urgent problem yet to be solved. It is worth noting that our candidate target gene has a well-documented role in the regulation of plaque stability (Table 2).

## Conclusion and outlook

The transition of an atherosclerotic plaque from stable to unstable is a multi-step process involving multiple cells. The purpose of this study was to investigate the regulatory effect of ncRNAs targeting endothelial cells, vascular smooth muscle cells, and immune cells on plaque stability. Inflammation, lipid metabolism, and oxidative stress pathway are also closely related to plaque stability. NcRNAs can stably exist in the plasma and other body fluids, and the use of ncRNA-targeted therapies has been widely recognized. The identification of unstable plaques at an early stage will be helpful for effective intervention in patients with CAD and significantly improve their prognosis 154. LncRNAs are potential regulators of the inflammatory response 155 and can regulate gene expression through epigenetic regulation, transcriptional regulation, post-transcriptional regulation, molecular sponge effects, and as molecular chaperones, thus playing a key role in the regulation of plaque stability^145^, ^156^. MiRNAs are expressed in the vascular system, participate in vascular inflammation and smooth muscle cell proliferation, and are now recognized as main regulators in the signaling pathways leading to plaque instability 147, 157.

Although lncRNAs transcripts are more numerous than protein-coding genes, their expression, function, and mechanism of action in the relevant cell types during development of the atherosclerotic plaque are unclear. Stable miRNAs in circulating blood can be used as endogenous disease biomarkers 158, 159. However, there are still some studies on how the release of miRNAs in the plasma reflects the dynamics and regulates the instability of carotid plaque. Some tools such as Tissue Atlas 157 and IMOTA (https://ccb-web.cs.uni-saarland.de/imota/) provide powerful means for the subsequent detection of the expression of miRNAs and their target genes in specific tissues. Some studies have demonstrated that lncRNAs combined with miRNAs play an important role in plaque stability, but whether this mechanism is universal has not been confirmed, and the relationship between lncRNAs and miRNAs remains to be further studied. To date, most studies have been carried out only *in vitro*; therefore, the role of ncRNAs and their targets needs to be further studied *in vivo*. In recent years, exosomes have been widely reported as a medium for extracellular transport. Some studies have shown that ncRNAs can be detected in extracellular transport vesicles in exosomes carrying biological media; therefore, it is thought that ncRNAs secreted to extracellular bodies are loaded by exosomes, and the special structure of the exosome's bilayer lipid membranes protect them from degradation, but the specific loading mechanism is not clear 160, 161. Whether exocrine ncRNAs can enter the cell cycle and play a role in regulating plaque stability still needs to be further explored. In addition, the development of methods for efficiently producing batches of exosomes and ncRNAs that can act on host cells remains a difficult problem. Importantly, discovering ncRNAs from scratch is very costly and often requires many sequencing efforts, without established standards for data analysis methods. In addition, ncRNAs undergo some modifications *in vivo*, such as methylation and splicing, making it more difficult to study the mechanism of plaque stability. With continuous improvements in RNA sequencing technology, more efficient RNA analysis methods can be applied to a small number of blood samples from specific patients 162. We believe that great progress will be made in exploring the utility of ncRNAs in the treatment of plaques in the future. Although our knowledge of the role of ncRNAs in plaque stability remains preliminary, this field is worthy of deeper exploration and greater research efforts.

## Figures and Tables

**Table 1:** Regulation of ncRNAs in the stability of atherosclerotic plaques

NcRNAs	Expression	Phenotype	Effect on plaque stability	Reference
LncR- TCONS_00024652	up regulation	promotes ECs proliferation and angiogenesis	increase	118
LncR-LINC00657	up regulation	promotes ECs angiogenesis	decrease	163
LncR-UC.98	down regulation	promotes ECs proliferation and adhesion	increase	115
miR-21	up regulation	regulates macrophage migration and adhesion	decrease	164-166
miR-200C	up regulation	induces ECs dysfunction to produce ROS	increase	167
miR-23a-5p	up regulation	cholesterol efflux reduces the formation of foam cells	decrease	114
miR-124-3p	down regulation	inhibits VSMCs collagen synthesis	decrease	121, 168
miR-10b	up regulation	induces apoptosis of macrophages	decrease	127
miR-124	up regulation	collagen synthesis disorder	decrease	169
miR-150	down regulation	increases VSMCs and collagen content, reduce macrophage infiltration and lipid accumulation	increase	128
miR-19b	up regulation	inhibits STAT3 transcriptional activity affects ECs proliferation, migration and angiogenesis	increase	170
miR-195	up regulation	inhibits the TLR2 inflammatory pathway	increase	129
miR-495	down regulation	increases of neovascularization after ischemia	increase	171
miR-455-3p	up regulation	regulates eNOS protein stability and NO production	decrease	112
miR-133a	up regulation	targets LDLRAP1 reduces lipid accumulation in VSMCs	increase	119
miR-210	up regulation	targets tumor suppressor gene APC regulation of VSMCs survival	increase	122
miR-181b	down regulation	regulates tissue inhibitor of metalloproteinase-3 expression	increase	172
miR-27b	down regulation	targets Naa15 regulates the activity of CCL20/CCR6 axis regulates ECs angiogenesis	increase	116
miR-494	down regulation	cholesterol levels and very low-density lipoprotein (VLDL) components fell	increase	171, 173
miR-24	down regulation	increases the expression of MMP-14 in macrophages	increase	130
miR-145	up regulation	adjusts the plasticity of VSMCs	increase	120
miR-33	down regulation	promotes the expression of ABCA1 and the clearance of cholesterol	increase	174

**Table 2:** The biomarkers of ncRNAs of the stability plaques detected in human serum of atherosclerosis

ncRNA	Expression	Fold change (FC)	Sample	Reference
miR-2110	up regulation	2	blood samples	114
miR-4439	down regulation	1.4	blood samples	114
miR-8084	down regulation	1.4	blood samples	114
miR-23a-5p	up regulation	4	blood samples	114
miR-320a	up regulation	4.2	blood samples	114, 175
miR-200C	up regulation	2	tissue sample	167
miR-210	up regulation	3.12	blood samples	146, 147
miR-21	up regulation	5.3	blood samples	146, 147, 164, 175
miR-34a	up regulation	2	tissue sample	146, 147, 176
miR-146a/b	up regulation	4.15	blood samples	146
miR-19b	up regulation	4	blood samples	147
miR-22	up regulation	-	blood samples	147
miR-143	up regulation	5	blood samples	147
miR-99b	up regulation	-	blood samples	177
miR-152	up regulation	4	blood samples	177
miR-422a	up regulation	3.8	blood samples	177
miR-145	up regulation	2.2	tissue sample	148
miR-100	up regulation	1.8	tissue sample	148
circR-284	up regulation	3	blood samples	151

## Abbreviations

CVDs: Cardiovascular diseases
CAD: Coronary artery disease
ACS: Acute coronary syndrome
ADP: Adenosine diphosphate
PAF: Platelet activating factor
NcRNAs: Non-coding RNAs
SiRNAs: Small interfering RNAs
EC: Endothelial cell
VSMCs: Vascular smooth muscle cells
PFKFB3: 6-phosphofructo-2-kinase/fructose-2,6-biphosphatase
WNT: Wingless-Related Integration Site
HUVEC: Human umbilical vein endothelial cell
VEGFR2: Vascular endothelial growth factor receptor 2
MMP-2: Matrix metalloproteinase-2
EndMT: Endothelial to mesenchymal transition
VCAM-1: Vascular adhesion molecule-1
CTRP9: C1q/TNF-related protein 9
PEDF: Pigment epithelium-derived factor
CHD: Coronary heart disease
P4HA1: Prolyl 4-hydroxylase subunit alpha-1
TLR2: Toll-like receptor 2
